# (1-Butyl-1,4-diaza­bicyclo­[2.2.2]octon-1-ium-κ*N* ^4^)trichloridocobalt(II)

**DOI:** 10.1107/S1600536809005893

**Published:** 2009-02-25

**Authors:** Sanchai Luachan, Bunlawee Yotnoi, Timothy J. Prior, Apinpus Rujiwatra

**Affiliations:** a Department of Chemistry, Faculty of Science, Chiang Mai University, Chiang Mai 50200, Thailand; b Department of Chemistry, University of Hull, Kingston upon Hull HU6 7RX, England

## Abstract

The title compound, [Co(C_10_H_21_N_2_)Cl_3_], was obtained as the by-product of the attempted synthesis of a cobalt sulfate framework using 1,4-diaza­bicyclo­[2.2.2]octane as an organic template. The asymmetric unit comprises two distinct mol­ecules, and in each, the cobalt(II) ions are tetra­hedrally coordinated by three chloride anions and one 1-butyl­diaza­bicyclo­[2.2.2]octan-1-ium cation. The organic ligands are generated *in situ*, and exhibit two forms differentiated by the eclipsed and staggered conformations of the butyl groups. These mol­ecules inter­act by way of C—H⋯Cl hydrogen bonds, forming a three-dimensional hydrogen-bonding array.

## Related literature

Examples of closely related structures are *N*-methyl-1,4-diaza­bicyclo­(2.2.2) octonium trichloro-aqua-nickel(II) (Ross & Stucky, 1969 ▶) and *N*,*N*′-dimethyl-1,4-diaza­niabicyclo­[2.2.2]octane tetra­chloro­cobaltate (C_8_H_18_N_2_)[CoCl_4_] (Qu & Sun, 2005 ▶). The organic cation in both structures do not coordinate to the cobalt ion but, in each case, the C—H⋯Cl hydrogen-bonding inter­actions are similar to those in the title compound. For hydrogen bonding in related structures, see: Bremner & Harrison (2003 ▶).
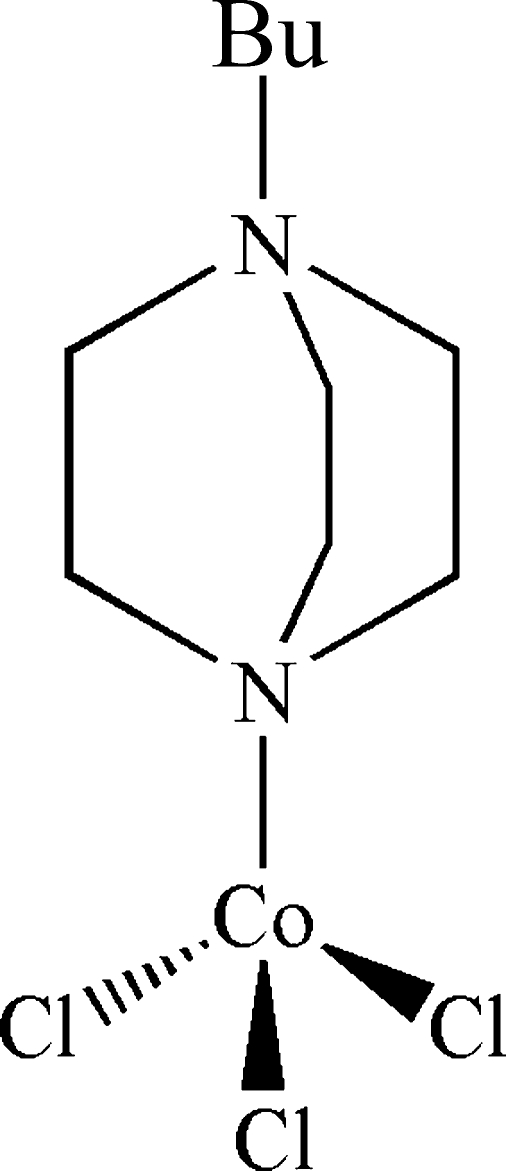


## Experimental

### 

#### Crystal data

[Co(C_10_H_21_N_2_)Cl_3_]
*M* *_r_* = 334.57
Monoclinic, 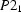

*a* = 8.379 (2) Å
*b* = 12.1090 (13) Å
*c* = 14.711 (4) Å
β = 91.683 (4)°
*V* = 1492.0 (6) Å^3^
*Z* = 4
Synchrotron radiation
λ = 0.69430 Å
μ = 1.67 mm^−1^
*T* = 120 K
0.12 × 0.02 × 0.02 mm

#### Data collection

Bruker D8 with APEXII detector diffractometer
Absorption correction: multi-scan (TWINABS; Bruker, 2004 ▶) *T*_min_ = 0.597, *T*_max_ = 0.746 (expected range = 0.774–0.967)
12848 measured reflections
8831 independent reflections
7018 reflections with *I* > 2σ(*I*)
*R*_int_ = 0.054

#### Refinement

*R*[*F* ^2^ > 2σ(*F* ^2^)] = 0.045
*wR*(*F* ^2^) = 0.098
*S* = 1.04
8831 reflections
292 parameters
1 restraint
H-atom parameters constrained
Δρ_max_ = 0.65 e Å^−3^
Δρ_min_ = −0.44 e Å^−3^
Absolute structure: Flack (1983 ▶), 3980 Friedel pairs
Flack parameter: 0.064 (17)

### 

Data collection: *APEX2* (Bruker, 2007 ▶); cell refinement: *APEX2*; data reduction: *TWINABS* (Bruker, 2004 ▶); program(s) used to solve structure: *SHELXS86* (Sheldrick, 2008 ▶); program(s) used to refine structure: *SHELXL97* (Sheldrick, 2008 ▶); molecular graphics: *DIAMOND* (Brandenburg & Putz, 2005 ▶); software used to prepare material for publication: *PLATON* (Spek, 2009 ▶).

## Supplementary Material

Crystal structure: contains datablocks I, global. DOI: 10.1107/S1600536809005893/lh2775sup1.cif

Structure factors: contains datablocks I. DOI: 10.1107/S1600536809005893/lh2775Isup2.hkl

Additional supplementary materials:  crystallographic information; 3D view; checkCIF report

**Fig. 5.**
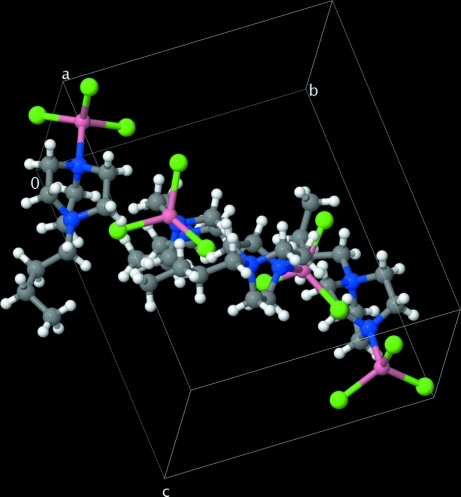
Molecular packing in unit cell.

Enhanced figure: interactive version of Fig. 5

## Figures and Tables

**Table 1:** Hydrogen-bond geometry (Å, °)

*D*—H⋯*A*	*D*—H	H⋯*A*	*D*⋯*A*	*D*—H⋯*A*
C2—H2*B*⋯Cl6^i^	0.99	2.66	3.567 (5)	153
C4—H4*A*⋯Cl1^ii^	0.99	2.66	3.511 (5)	145
C6—H6*B*⋯Cl3^ii^	0.99	2.69	3.606 (5)	154
C7—H7*B*⋯Cl3^iii^	0.99	2.80	3.729 (5)	157
C12—H12*B*⋯Cl5^iv^	0.99	2.62	3.485 (4)	146
C14—H14*A*⋯Cl6^iv^	0.99	2.75	3.567 (5)	140
C16—H16*A*⋯Cl1^v^	0.99	2.60	3.548 (4)	161
C16—H16*B*⋯Cl5^v^	0.99	2.81	3.739 (4)	156

Symmetry codes: (i) 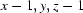; (ii) 
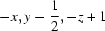
; (iii) 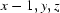; (iv) 
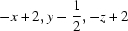
; (v) 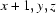.

**Fig. 1.**
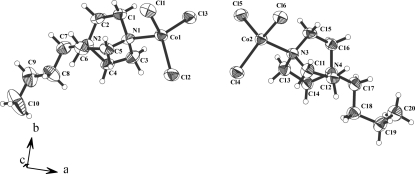
View of the title compound with the atom numbering scheme. Displacement ellipsoids for non-H atoms are drawn at the 70% probability level.

**Fig. 2.**
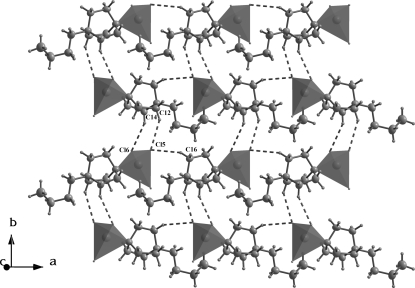
View of the A sheet along the ab plane with the hydrogen bonding atoms indicated.

**Fig. 3.**
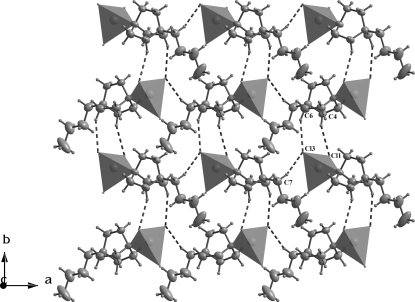
View of the B sheet along the ab plane with the hydrogen bonding atoms indicated.

**Fig. 4.**
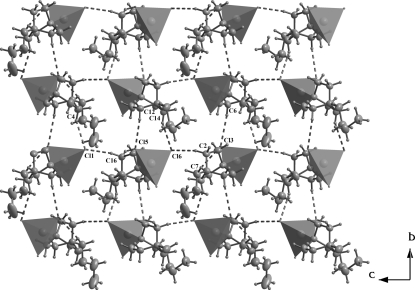
The packing of A and B sheets along c in ABAB fashion.

## supplementary crystallographic information

### Comment

The crystals of Co(C_10_H_21_N_2_)Cl_3_ (I) were unintentionally obtained as a by-product from the hydrothermal reaction between cobalt(II) sulfate heptahydrate and 1,4-diazabicyclo[2.2.2]octane in a water/butan-1-ol mixture. The *N*-butyl-1,4-diazabicyclo[2.2.2]octanium ligand was presumably generated *in situ* under acidic conditions. The structure of I is built up from two distinct [Co(C_10_H_21_N_2_)Cl_3_] molecules as shown in Fig. 1. They are different in the spatial orientation of the butyl group of the *N*-butyl-1,4-diazabicyclo[2.2.2]octanium ligand, one of which is in the eclipsed conformation (A) and the other is in the staggered conformation (B). The A molecules are connected by the C—H···Cl hydrogen bonding interactions to form a two-dimensional A sheet in the *ab* plane (Fig. 2), whereas the B molecules form the B sheet also in the*ab* plane using similar C—H···Cl hydrogen bonding interactions (Fig. 3). The A and B sheets are then regularly alternated in the ABAB fashion, and linked by way of also the C—H···Cl hydrogen bonding interactions along *c* to give the infinite three-dimensional hydrogen bonding array (Fig. 4).

The hydrogen bond geometries found in I (H···Cl, 2.62–2.81 Å; C···Cl, 3.485 (4)–3.739 (4) Å; C—H···Cl, 140.00–164.00°) are well comparable to those found in related structures, *e.g.* (C_6_H_14_N_2_)[CoCl_4_] (Bremner & Harrison, 2003) and (C_8_H_18_N_2_)[CoCl_4_] (Qu & Sun, 2005).

### Experimental

Crystals of I were obtained as a by-product from the hydrothermal reaction of cobalt(II) sulfate heptahydrate, 1,4-diazabicyclo[2.2.2]octane and hydrochloric acid in a water/butan-1-ol mixture at 453 K for 120 h.

### Refinement

H atoms were placed in calcluated positions with C-H = 0.99Å or 0.98Å for methyl H atoms and were included in the refinement in a riding-model approximation with U_iso_(H) = 1.2U_eq_(C) or 1.5U_eq_(C) for methyl H atoms.

The examined crystal was found to be twinned, composing of two crystal components which were miss-set by about two degrees. The crystal was therefore treated as a twin and the two components integrated separately using the same unit cell. Both components were used for the structure refinement and the twin fraction was found to be 0.698:0.302 (1).

Three alerts from checkCIF:

PLAT220_ALERT_2_C

PLAT222_ALERT_2_C

The rather weak van der Waals interactions involving the *n*-butyl chains mean there is considerable freedom for these carbon and hydrogen atoms to vibrate. The slightly enlarged displacement parameters observed are entirely expected on chemical grounds.

PLAT341_ALERT_3_C

The calculated estimated standard uncertainties associated with the unit-cell parameters are faithfully reproduced from the Bruker APEXII suite (Bruker, 2004). All observed data were used in their calculation. These give rise to moderate precision in the C—C bonds. To some extent this is a consequence of the integration procedure which uses two twin components - deconvolution of the low angle components is problematic as the two componenets are miss-set by approximately 2°.

### Crystal data

**Table:**

[Co(C_10_H_21_N_2_)Cl_3_]	*F*(000) = 692
*M**_r_* = 334.57	*D*_x_ = 1.490 Mg m^−^^3^
Monoclinic, *P*2_1_	Synchrotron radiation, λ = 0.69430 Å
Hall symbol: P 2yb	Cell parameters from 12848 reflections
*a* = 8.379 (2) Å	θ = 1.4–30.7°
*b* = 12.1090 (13) Å	µ = 1.67 mm^−^^1^
*c* = 14.711 (4) Å	*T* = 120 K
β = 91.683 (4)°	Needle, blue
*V* = 1492.0 (6) Å^3^	0.12 × 0.02 × 0.02 mm
*Z* = 4	

### Data collection

**Table:**

Bruker D8 with APEXII detector diffractometer	8831 independent reflections
Radiation source: Daresbury SRS, UK	7018 reflections with *I* > 2σ(*I*)
silicon 111	*R*_int_ = 0.054
ω scans	θ_max_ = 30.7°, θ_min_ = 1.4°
Absorption correction: multi-scan (TWINABS; Bruker, 2004)	*h* = −12→12
*T*_min_ = 0.597, *T*_max_ = 0.746	*k* = −17→17
12848 measured reflections	*l* = −20→20

### Refinement

**Table:**

Refinement on *F*^2^	Secondary atom site location: difference Fourier map
Least-squares matrix: full	Hydrogen site location: inferred from neighbouring sites
*R*[*F*^2^ > 2σ(*F*^2^)] = 0.045	H-atom parameters constrained
*wR*(*F*^2^) = 0.098	*w* = 1/[σ^2^(*F*_o_^2^) + (0.0352*P*)^2^ + 0.2945*P*] where *P* = (*F*_o_^2^ + 2*F*_c_^2^)/3
*S* = 1.04	(Δ/σ)_max_ = 0.001
8831 reflections	Δρ_max_ = 0.65 e Å^−^^3^
292 parameters	Δρ_min_ = −0.43 e Å^−^^3^
1 restraint	Absolute structure: Flack (1983), 3980 Friedel pairs
Primary atom site location: structure-invariant direct methods	Flack parameter: 0.064 (17)

### Special details

**Table:**

Geometry. All e.s.d.'s (except the e.s.d. in the dihedral angle between two l.s. planes) are estimated using the full covariance matrix. The cell e.s.d.'s are taken into account individually in the estimation of e.s.d.'s in distances, angles and torsion angles; correlations between e.s.d.'s in cell parameters are only used when they are defined by crystal symmetry. An approximate (isotropic) treatment of cell e.s.d.'s is used for estimating e.s.d.'s involving l.s. planes.
Refinement. Refinement of *F*^2^ against ALL reflections. The weighted *R*-factor *wR* and goodness of fit *S* are based on *F*^2^, conventional *R*-factors *R* are based on *F*, with *F* set to zero for negative *F*^2^. The threshold expression of *F*^2^ > σ(*F*^2^) is used only for calculating *R*-factors(gt) *etc*. and is not relevant to the choice of reflections for refinement. *R*-factors based on *F*^2^ are statistically about twice as large as those based on *F*, and *R*- factors based on ALL data will be even larger.

### Fractional atomic coordinates and isotropic or equivalent isotropic displacement parameters (Å^2^)

**Table:**

	*x*	*y*	*z*	*U*_iso_*/*U*_eq_	
Co1	0.17969 (6)	0.70552 (4)	0.56075 (3)	0.02973 (12)	
Cl1	0.08844 (13)	0.78922 (10)	0.68543 (6)	0.0382 (2)	
Cl2	0.28560 (14)	0.53800 (9)	0.58911 (8)	0.0416 (2)	
Cl3	0.32696 (13)	0.81305 (9)	0.47062 (7)	0.0373 (2)	
N1	−0.0308 (4)	0.6782 (3)	0.48346 (19)	0.0264 (7)	
N2	−0.2901 (4)	0.6376 (3)	0.3900 (2)	0.0278 (7)	
C1	−0.1094 (5)	0.7827 (4)	0.4539 (3)	0.0304 (8)	
H1A	−0.0310	0.8306	0.4240	0.037*	
H1B	−0.1485	0.8224	0.5077	0.037*	
C2	−0.2502 (5)	0.7587 (3)	0.3875 (3)	0.0296 (9)	
H2A	−0.3443	0.8026	0.4047	0.036*	
H2B	−0.2216	0.7801	0.3251	0.036*	
C3	0.0056 (5)	0.6132 (4)	0.4005 (3)	0.0327 (9)	
H3A	0.0741	0.5495	0.4175	0.039*	
H3B	0.0647	0.6601	0.3578	0.039*	
C4	−0.1487 (5)	0.5719 (4)	0.3539 (3)	0.0319 (9)	
H4A	−0.1630	0.4923	0.3665	0.038*	
H4B	−0.1430	0.5818	0.2872	0.038*	
C5	−0.1470 (5)	0.6141 (4)	0.5373 (3)	0.0318 (8)	
H5A	−0.1588	0.6499	0.5972	0.038*	
H5B	−0.1048	0.5386	0.5480	0.038*	
C6	−0.3093 (5)	0.6065 (4)	0.4892 (2)	0.0315 (8)	
H6A	−0.3853	0.6575	0.5179	0.038*	
H6B	−0.3516	0.5305	0.4937	0.038*	
C7	−0.4381 (6)	0.6160 (4)	0.3329 (3)	0.0351 (9)	
H7A	−0.4196	0.6415	0.2701	0.042*	
H7B	−0.5264	0.6607	0.3569	0.042*	
C8	−0.4898 (7)	0.4966 (4)	0.3294 (3)	0.0477 (13)	
H8A	−0.5210	0.4732	0.3909	0.057*	
H8B	−0.3982	0.4504	0.3118	0.057*	
C9	−0.6289 (7)	0.4765 (5)	0.2626 (3)	0.0541 (15)	
H9A	−0.7187	0.5257	0.2779	0.065*	
H9B	−0.5958	0.4951	0.2004	0.065*	
C10	−0.6843 (9)	0.3581 (7)	0.2648 (4)	0.086 (3)	
H10A	−0.5968	0.3094	0.2473	0.129*	
H10B	−0.7752	0.3484	0.2221	0.129*	
H10C	−0.7167	0.3393	0.3264	0.129*	
Co2	0.86290 (6)	0.69012 (4)	1.06702 (3)	0.02719 (12)	
Cl4	0.74916 (13)	0.52684 (9)	1.09830 (7)	0.0360 (2)	
Cl5	0.70380 (13)	0.79352 (9)	0.97538 (7)	0.0361 (2)	
Cl6	0.97391 (13)	0.78267 (9)	1.18605 (6)	0.0331 (2)	
N3	1.0632 (4)	0.6586 (3)	0.98991 (19)	0.0256 (7)	
N4	1.3114 (4)	0.6133 (3)	0.8981 (2)	0.0266 (7)	
C11	1.1771 (5)	0.5881 (4)	1.0443 (2)	0.0335 (9)	
H11A	1.1303	0.5138	1.0523	0.040*	
H11B	1.1955	0.6211	1.1052	0.040*	
C12	1.3367 (5)	0.5778 (3)	0.9963 (2)	0.0290 (8)	
H12A	1.4181	0.6254	1.0268	0.035*	
H12B	1.3748	0.5005	0.9990	0.035*	
C13	1.0170 (5)	0.5989 (4)	0.9044 (3)	0.0323 (9)	
H13A	0.9572	0.6493	0.8628	0.039*	
H13B	0.9464	0.5360	0.9186	0.039*	
C14	1.1659 (5)	0.5563 (4)	0.8579 (2)	0.0303 (9)	
H14A	1.1755	0.4755	0.8667	0.036*	
H14B	1.1569	0.5712	0.7918	0.036*	
C15	1.1471 (5)	0.7610 (3)	0.9650 (3)	0.0314 (8)	
H15A	1.1927	0.7963	1.0206	0.038*	
H15B	1.0701	0.8131	0.9361	0.038*	
C16	1.2827 (5)	0.7362 (3)	0.8985 (2)	0.0279 (8)	
H16A	1.2516	0.7618	0.8366	0.033*	
H16B	1.3814	0.7755	0.9183	0.033*	
C17	1.4589 (5)	0.5863 (4)	0.8441 (3)	0.0345 (9)	
H17A	1.5547	0.6134	0.8782	0.041*	
H17B	1.4522	0.6265	0.7855	0.041*	
C18	1.4796 (5)	0.4631 (4)	0.8249 (3)	0.0348 (9)	
H18A	1.4422	0.4199	0.8773	0.042*	
H18B	1.4131	0.4424	0.7708	0.042*	
C19	1.6527 (5)	0.4346 (4)	0.8082 (3)	0.0385 (10)	
H19A	1.6602	0.3550	0.7935	0.046*	
H19B	1.7167	0.4477	0.8648	0.046*	
C20	1.7244 (6)	0.5012 (4)	0.7312 (3)	0.0417 (11)	
H20A	1.6572	0.4932	0.6760	0.062*	
H20B	1.8321	0.4739	0.7197	0.062*	
H20C	1.7300	0.5793	0.7485	0.062*	

### Atomic displacement parameters (Å^2^)

**Table:**

	*U*^11^	*U*^22^	*U*^33^	*U*^12^	*U*^13^	*U*^23^
Co1	0.0326 (3)	0.0238 (3)	0.0327 (2)	−0.0009 (2)	−0.0002 (2)	0.0029 (2)
Cl1	0.0502 (6)	0.0355 (6)	0.0288 (4)	−0.0037 (5)	0.0015 (4)	−0.0005 (4)
Cl2	0.0429 (6)	0.0268 (5)	0.0546 (6)	0.0019 (5)	−0.0071 (5)	0.0055 (4)
Cl3	0.0385 (5)	0.0285 (5)	0.0454 (5)	−0.0021 (4)	0.0099 (4)	0.0031 (4)
N1	0.0319 (17)	0.0216 (17)	0.0260 (13)	0.0059 (13)	0.0046 (12)	0.0006 (12)
N2	0.0305 (18)	0.0259 (18)	0.0271 (15)	0.0015 (14)	0.0002 (13)	−0.0009 (12)
C1	0.034 (2)	0.023 (2)	0.0347 (18)	0.0029 (17)	0.0007 (15)	0.0025 (15)
C2	0.036 (2)	0.024 (2)	0.0297 (18)	0.0071 (16)	0.0058 (16)	−0.0001 (14)
C3	0.035 (2)	0.036 (2)	0.0273 (17)	0.0080 (18)	0.0036 (15)	−0.0027 (15)
C4	0.035 (2)	0.026 (2)	0.0349 (19)	0.0051 (17)	0.0052 (16)	−0.0050 (15)
C5	0.040 (2)	0.028 (2)	0.0277 (17)	−0.0031 (18)	0.0017 (15)	0.0001 (14)
C6	0.041 (2)	0.031 (2)	0.0230 (16)	−0.0036 (19)	0.0033 (15)	0.0048 (14)
C7	0.040 (2)	0.036 (2)	0.0294 (18)	0.0053 (19)	0.0000 (16)	−0.0029 (15)
C8	0.058 (3)	0.045 (3)	0.040 (2)	−0.015 (2)	−0.005 (2)	−0.0049 (19)
C9	0.045 (3)	0.077 (4)	0.040 (2)	−0.015 (3)	0.005 (2)	−0.018 (2)
C10	0.086 (5)	0.118 (7)	0.054 (3)	−0.066 (5)	0.020 (3)	−0.032 (4)
Co2	0.0293 (3)	0.0241 (3)	0.0285 (2)	−0.0002 (2)	0.00579 (18)	−0.0004 (2)
Cl4	0.0406 (6)	0.0265 (5)	0.0415 (5)	−0.0042 (4)	0.0101 (4)	0.0004 (4)
Cl5	0.0366 (5)	0.0301 (6)	0.0414 (5)	0.0042 (4)	−0.0033 (4)	−0.0018 (4)
Cl6	0.0415 (5)	0.0306 (5)	0.0276 (4)	−0.0026 (5)	0.0055 (4)	−0.0014 (4)
N3	0.0284 (17)	0.0228 (17)	0.0258 (14)	−0.0007 (13)	0.0045 (12)	−0.0004 (11)
N4	0.0296 (17)	0.0228 (17)	0.0276 (15)	−0.0018 (14)	0.0030 (12)	−0.0029 (12)
C11	0.033 (2)	0.041 (2)	0.0269 (17)	0.0037 (19)	0.0052 (15)	0.0063 (16)
C12	0.034 (2)	0.0229 (19)	0.0305 (17)	0.0007 (16)	0.0021 (15)	0.0017 (14)
C13	0.028 (2)	0.039 (2)	0.0296 (18)	−0.0032 (18)	0.0005 (15)	−0.0075 (16)
C14	0.028 (2)	0.032 (2)	0.0308 (18)	−0.0036 (17)	0.0071 (15)	−0.0063 (15)
C15	0.040 (2)	0.021 (2)	0.0335 (18)	−0.0005 (17)	0.0088 (17)	0.0034 (14)
C16	0.030 (2)	0.026 (2)	0.0269 (17)	−0.0035 (15)	0.0037 (15)	0.0036 (14)
C17	0.035 (2)	0.035 (2)	0.035 (2)	−0.0053 (19)	0.0128 (17)	−0.0069 (17)
C18	0.037 (2)	0.031 (2)	0.036 (2)	−0.0011 (18)	0.0072 (17)	−0.0030 (16)
C19	0.034 (2)	0.046 (3)	0.036 (2)	0.006 (2)	0.0090 (18)	0.0050 (19)
C20	0.045 (3)	0.046 (3)	0.035 (2)	−0.003 (2)	0.0141 (19)	−0.0008 (19)

### Geometric parameters (Å, °)

**Table:**

Co1—N1	2.096 (3)	Co2—N3	2.088 (3)
Co1—Cl2	2.2483 (13)	Co2—Cl4	2.2482 (12)
Co1—Cl1	2.2491 (12)	Co2—Cl5	2.2487 (12)
Co1—Cl3	2.2521 (11)	Co2—Cl6	2.2564 (11)
N1—C1	1.486 (5)	N3—C15	1.477 (5)
N1—C3	1.491 (5)	N3—C13	1.493 (5)
N1—C5	1.491 (5)	N3—C11	1.495 (5)
N2—C7	1.500 (6)	N4—C14	1.507 (5)
N2—C2	1.505 (5)	N4—C16	1.508 (5)
N2—C6	1.520 (5)	N4—C12	1.516 (5)
N2—C4	1.536 (5)	N4—C17	1.524 (5)
C1—C2	1.537 (6)	C11—C12	1.536 (6)
C1—H1A	0.9900	C11—H11A	0.9900
C1—H1B	0.9900	C11—H11B	0.9900
C2—H2A	0.9900	C12—H12A	0.9900
C2—H2B	0.9900	C12—H12B	0.9900
C3—C4	1.530 (6)	C13—C14	1.530 (5)
C3—H3A	0.9900	C13—H13A	0.9900
C3—H3B	0.9900	C13—H13B	0.9900
C4—H4A	0.9900	C14—H14A	0.9900
C4—H4B	0.9900	C14—H14B	0.9900
C5—C6	1.517 (6)	C15—C16	1.550 (5)
C5—H5A	0.9900	C15—H15A	0.9900
C5—H5B	0.9900	C15—H15B	0.9900
C6—H6A	0.9900	C16—H16A	0.9900
C6—H6B	0.9900	C16—H16B	0.9900
C7—C8	1.510 (7)	C17—C18	1.530 (6)
C7—H7A	0.9900	C17—H17A	0.9900
C7—H7B	0.9900	C17—H17B	0.9900
C8—C9	1.522 (7)	C18—C19	1.518 (6)
C8—H8A	0.9900	C18—H18A	0.9900
C8—H8B	0.9900	C18—H18B	0.9900
C9—C10	1.508 (9)	C19—C20	1.528 (6)
C9—H9A	0.9900	C19—H19A	0.9900
C9—H9B	0.9900	C19—H19B	0.9900
C10—H10A	0.9800	C20—H20A	0.9800
C10—H10B	0.9800	C20—H20B	0.9800
C10—H10C	0.9800	C20—H20C	0.9800
			
N1—Co1—Cl2	106.21 (10)	N3—Co2—Cl4	107.62 (10)
N1—Co1—Cl1	102.29 (9)	N3—Co2—Cl5	104.35 (9)
Cl2—Co1—Cl1	113.39 (5)	Cl4—Co2—Cl5	111.41 (5)
N1—Co1—Cl3	103.81 (9)	N3—Co2—Cl6	101.11 (10)
Cl2—Co1—Cl3	114.25 (5)	Cl4—Co2—Cl6	116.46 (4)
Cl1—Co1—Cl3	115.14 (5)	Cl5—Co2—Cl6	114.35 (5)
C1—N1—C3	108.0 (3)	C15—N3—C13	108.1 (3)
C1—N1—C5	107.9 (3)	C15—N3—C11	108.1 (3)
C3—N1—C5	108.2 (3)	C13—N3—C11	108.7 (3)
C1—N1—Co1	112.5 (2)	C15—N3—Co2	112.2 (2)
C3—N1—Co1	109.8 (2)	C13—N3—Co2	110.7 (2)
C5—N1—Co1	110.3 (2)	C11—N3—Co2	108.9 (2)
C7—N2—C2	109.7 (3)	C14—N4—C16	109.0 (3)
C7—N2—C6	112.7 (3)	C14—N4—C12	109.5 (3)
C2—N2—C6	107.1 (3)	C16—N4—C12	107.1 (3)
C7—N2—C4	110.5 (3)	C14—N4—C17	110.9 (3)
C2—N2—C4	108.8 (3)	C16—N4—C17	110.2 (3)
C6—N2—C4	108.0 (3)	C12—N4—C17	110.1 (3)
N1—C1—C2	110.5 (3)	N3—C11—C12	110.6 (3)
N1—C1—H1A	109.5	N3—C11—H11A	109.5
C2—C1—H1A	109.5	C12—C11—H11A	109.5
N1—C1—H1B	109.5	N3—C11—H11B	109.5
C2—C1—H1B	109.5	C12—C11—H11B	109.5
H1A—C1—H1B	108.1	H11A—C11—H11B	108.1
N2—C2—C1	109.6 (3)	N4—C12—C11	108.4 (3)
N2—C2—H2A	109.8	N4—C12—H12A	110.0
C1—C2—H2A	109.8	C11—C12—H12A	110.0
N2—C2—H2B	109.8	N4—C12—H12B	110.0
C1—C2—H2B	109.8	C11—C12—H12B	110.0
H2A—C2—H2B	108.2	H12A—C12—H12B	108.4
N1—C3—C4	110.4 (3)	N3—C13—C14	110.2 (3)
N1—C3—H3A	109.6	N3—C13—H13A	109.6
C4—C3—H3A	109.6	C14—C13—H13A	109.6
N1—C3—H3B	109.6	N3—C13—H13B	109.6
C4—C3—H3B	109.6	C14—C13—H13B	109.6
H3A—C3—H3B	108.1	H13A—C13—H13B	108.1
C3—C4—N2	109.0 (3)	N4—C14—C13	109.4 (3)
C3—C4—H4A	109.9	N4—C14—H14A	109.8
N2—C4—H4A	109.9	C13—C14—H14A	109.8
C3—C4—H4B	109.9	N4—C14—H14B	109.8
N2—C4—H4B	109.9	C13—C14—H14B	109.8
H4A—C4—H4B	108.3	H14A—C14—H14B	108.3
N1—C5—C6	112.0 (3)	N3—C15—C16	111.0 (3)
N1—C5—H5A	109.2	N3—C15—H15A	109.4
C6—C5—H5A	109.2	C16—C15—H15A	109.4
N1—C5—H5B	109.2	N3—C15—H15B	109.4
C6—C5—H5B	109.2	C16—C15—H15B	109.4
H5A—C5—H5B	107.9	H15A—C15—H15B	108.0
C5—C6—N2	108.3 (3)	N4—C16—C15	108.3 (3)
C5—C6—H6A	110.0	N4—C16—H16A	110.0
N2—C6—H6A	110.0	C15—C16—H16A	110.0
C5—C6—H6B	110.0	N4—C16—H16B	110.0
N2—C6—H6B	110.0	C15—C16—H16B	110.0
H6A—C6—H6B	108.4	H16A—C16—H16B	108.4
N2—C7—C8	114.7 (4)	N4—C17—C18	113.8 (3)
N2—C7—H7A	108.6	N4—C17—H17A	108.8
C8—C7—H7A	108.6	C18—C17—H17A	108.8
N2—C7—H7B	108.6	N4—C17—H17B	108.8
C8—C7—H7B	108.6	C18—C17—H17B	108.8
H7A—C7—H7B	107.6	H17A—C17—H17B	107.7
C7—C8—C9	112.8 (5)	C19—C18—C17	111.5 (4)
C7—C8—H8A	109.0	C19—C18—H18A	109.3
C9—C8—H8A	109.0	C17—C18—H18A	109.3
C7—C8—H8B	109.0	C19—C18—H18B	109.3
C9—C8—H8B	109.0	C17—C18—H18B	109.3
H8A—C8—H8B	107.8	H18A—C18—H18B	108.0
C10—C9—C8	111.6 (6)	C18—C19—C20	113.4 (4)
C10—C9—H9A	109.3	C18—C19—H19A	108.9
C8—C9—H9A	109.3	C20—C19—H19A	108.9
C10—C9—H9B	109.3	C18—C19—H19B	108.9
C8—C9—H9B	109.3	C20—C19—H19B	108.9
H9A—C9—H9B	108.0	H19A—C19—H19B	107.7
C9—C10—H10A	109.5	C19—C20—H20A	109.5
C9—C10—H10B	109.5	C19—C20—H20B	109.5
H10A—C10—H10B	109.5	H20A—C20—H20B	109.5
C9—C10—H10C	109.5	C19—C20—H20C	109.5
H10A—C10—H10C	109.5	H20A—C20—H20C	109.5
H10B—C10—H10C	109.5	H20B—C20—H20C	109.5

### Hydrogen-bond geometry (Å, °)

**Table:**

*D*—H···*A*	*D*—H	H···*A*	*D*···*A*	*D*—H···*A*
C2—H2B···Cl6^i^	0.99	2.66	3.567 (5)	153
C4—H4A···Cl1^ii^	0.99	2.66	3.511 (5)	145
C6—H6B···Cl3^ii^	0.99	2.69	3.606 (5)	154
C7—H7B···Cl3^iii^	0.99	2.80	3.729 (5)	157
C12—H12B···Cl5^iv^	0.99	2.62	3.485 (4)	146
C14—H14A···Cl6^iv^	0.99	2.75	3.567 (5)	140
C16—H16A···Cl1^v^	0.99	2.60	3.548 (4)	161
C16—H16B···Cl5^v^	0.99	2.81	3.739 (4)	156

Symmetry codes: (i) *x*−1, *y*, *z*−1; (ii) −*x*, *y*−1/2, −*z*+1; (iii) *x*−1, *y*, *z*; (iv) −*x*+2, *y*−1/2, −*z*+2; (v) *x*+1, *y*, *z*.
